# Photobleaching Response of Different Sources of Chromophoric Dissolved Organic Matter Exposed to Natural Solar Radiation Using Absorption and Excitation–Emission Matrix Spectra

**DOI:** 10.1371/journal.pone.0077515

**Published:** 2013-10-25

**Authors:** Yunlin Zhang, Xiaohan Liu, Christopher L. Osburn, Mingzhu Wang, Boqiang Qin, Yongqiang Zhou

**Affiliations:** 1 Taihu Lake Laboratory Ecosystem Research Station, State Key Laboratory of Lake Science and Environment, Nanjing Institute of Geography and Limnology, Chinese Academy of Sciences, Nanjing, China; 2 University of Chinese Academy of Sciences, Beijing, China; 3 Department of Marine, Earth, and Atmospheric Sciences, North Carolina State University, Raleigh, North Carolina, United States of America; University of California, Merced, United States of America

## Abstract

CDOM biogeochemical cycle is driven by several physical and biological processes such as river input, biogeneration and photobleaching that act as primary sinks and sources of CDOM. Watershed-derived allochthonous (WDA) and phytoplankton-derived autochthonous (PDA) CDOM were exposed to 9 days of natural solar radiation to assess the photobleaching response of different CDOM sources, using absorption and fluorescence (excitation-emission matrix) spectroscopy. Our results showed a marked decrease in total dissolved nitrogen (TDN) concentration under natural sunlight exposure for both WDA and PDA CDOM, indicating photoproduction of ammonium from TDN. In contrast, photobleaching caused a marked increase in total dissolved phosphorus (TDP) concentration for both WDA and PDA CDOM. Thus TDN∶TDP ratios decreased significantly both for WDA and PDA CDOM, which partially explained the seasonal dynamic of TDN∶TDP ratio in Lake Taihu. Photobleaching rate of CDOM absorption *a*(254), was 0.032 m/MJ for WDA CDOM and 0.051 m/MJ for PDA CDOM from days 0–9, indicating that phototransformations were initially more rapid for the newly produced CDOM from phytoplankton than for the river CDOM. Extrapolation of these values to the field indicated that 3.9%–5.1% CDOM at the water surface was photobleached and mineralized every day in summer in Lake Taihu. Photobleaching caused the increase of spectral slope, spectral slope ratio and molecular size, indicating the CDOM mean molecular weight decrease which was favorable to further microbial degradation of mineralization. Three fluorescent components were validated in parallel factor analysis models calculated separately for WDA and PDA CDOM. Our study suggests that the humic-like fluorescence materials could be rapidly and easily photobleached for WDA and PDA CDOM, but the protein-like fluorescence materials was not photobleached and even increased from the transformation of the humic-like fluorescence substance to the protein-like fluorescence substance. Photobleaching was an important driver of CDOM and nutrients biogeochemistry in lake water.

## Introduction

Chromophoric dissolved organic matter (CDOM), a major reservoir of organic carbon in aquatic environments, plays a central role in many physical, chemical and biological processes in aquatic ecosystems, including inhibiting attenuation of harmful ultraviolet radiation [1], affecting carbon budgets, nutrient availability and ecosystem productivity [2]–[4], disturbing water color parameters and estimates of primary production from remote sensing reflectances [5], [6]. Large and shallow eutrophic lake ecosystems, such as Lake Taihu, China, and its surrounding rivers, are complex and dynamic environments of intense CDOM cycling in which the quantity, quality and distribution of CDOM reflect a balance between inputs and decomposition. In these systems, the sources of CDOM include the terrestrial river inputs and seasonal *in situ* production from frequent algal bloom and dense macrophytes [7]–[9].

The residence time, cycling and fate of CDOM in aquatic environments are regulated primarily by biological degradation and photoinduced degradation. Biological degradation is, in turn, mediated primarily by bacteria that use the labile CDOM fraction for growth and respiration. Photoinduced degradation of CDOM during solar exposure generates a variety of photoproducts, including: (i) reactive oxygen species [10], (ii) atmospherically important trace gases, such as CO, CO_2_ and COS that affect global carbon cycling [11]–[13], (iii) low molecular weight labile carbonyl compounds that are readily available for consumption by microbial communities [14], and (iv) nutrients needed to support phytoplankton primary production [15]. The photoreactivity of CDOM is strongly influenced by its origin and chemical composition [16]. Recently, studies have evaluated the changes in the absorption and fluorescence characteristics of CDOM during photobleaching, using the allochthonous riverine CDOM or biological autochthonous CDOM under carefully controlled conditions [17]–[19]. There are only a few systematic studies comparing the photobleaching response and CDOM compositional changes of different sources of CDOM exposed to the natural sunlight [17]. In addition, although photobleaching results in the partial remineralization of CDOM in lakes, thereby affecting nutrient dynamics in the euphotic zone, there is little knowledge about the photoinduced transformations that mediate these dynamics in lakes [20].

In eutrophic shallow lakes, phytoplankton degradation may provide an important source of CDOM [7], [8]. Thus the photomineralization of phytoplankton-derived autochthonous (PDA) CDOM may be an important source of carbon and nutrient regeneration for bacteria and phytoplankton. The objectives of this study were to evaluate and compare the photoinduced cycling of watershed-derived allochthonous (WDA) and PDA CDOM with regard to absorption, fluorescence properties, overall changes in composition, and nutrients.

## Materials and Methods

### Ethics Statement

No permits were required for the field studies, because the location was not privately-owned or protected, and the field studies did not involve endangered or protected species.

### CDOM sample preparation

**Figure 1:**
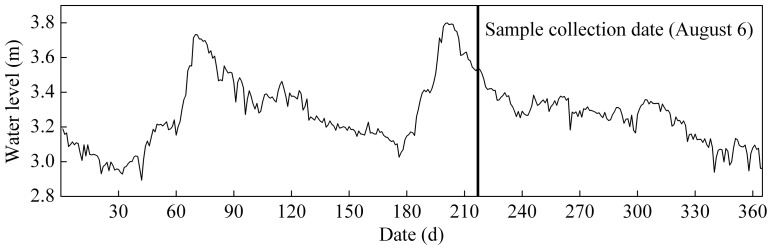
Daily variation of water level in Lake Taihu in 2010.

To determine the bleaching dynamics of different sources of CDOM exposed to natural radiation, we used WDA CDOM and PDA CDOM. Water for preparation of the WDA sample was collected on 6 August, 2010 from the entrance of the Wutangmen river into Meiliang Bay, Lake Taihu, China's third largest lake, (Lake Taihu means “large lake” in Mandarin). Sample collection was during the flood season (Fig. 1), indicating that the CDOM in the river was of the watershed-derived allochthonous variety [21]. The catchment area of the Wutangmen river is dominated by agriculture, urban development and wetlands. The 2.5 L sample was taken at 0.5 m below the surface of the water using a plexiglass sampler (Institute of Hydrobiology, Chinese Academy of Sciences).

Water for preparation of the PDA CDOM sample was collected on 4 August, 2010, from the littoral region of Meiliang Bay, in Lake Taihu, during a *Microcystis* algal bloom. The lake is highly eutrophic, and has experienced significant increases in nutrient loading and accelerated eutrophication in recent decades, with the TN and TP concentrations exceeding 5.0 mg/L and 0.11 mg/L, respectively [22]–[24]. The 20 L water sample was collected. The sample was diluted and cleaned using 10 L Milli-Q water, and then centrifuged at 5000 rpm for 15 min to separate the phytoplankton-derived particulate matter from the CDOM-containing lake water. The particulate matter was resuspended in 20 L of Milli-Q water, and the resulting suspension was degraded in the dark for 3 days at 25°C to produce the PDA CDOM.

Water samples from the river and phytoplankton degradation were initially filtered through a 47-mm diameter Whatman fiberglass GF/F filter combusted at 450°C, and then refiltered through a 25-mm diameter Millipore 0.22-µm porosity cellulose filter rinsed with Milli-Q water to remove the particles and bacteria following previous bleaching experiments [16], [17], [19], and thus obtain the WDA and PDA CDOM samples. This procedure removes >99.5% of bacteria, as determined by flow cytometry [25] thus minimizing the bacterial removal of CDOM.

### CDOM bleaching experiment

To assess the photobleaching response of different sources of CDOM, the WDA and PDA CDOM samples were exposed to natural solar radiation in the littoral zone of Meiliang Bay, Lake Taihu, at the Taihu Lake Laboratory Ecosystem Research (TLLER) station, Chinese Academy of Sciences, located at 31°25.42′, 120°12.57′. The bleaching experiment lasted 9 days, and samples were collected on 9 occasions: days 0–7 and 9.

Clear 60 ml quartz tubes were prepared by soaked in 10% nitric acid for 24 h, and then rinsed repeatedly with distilled water. The tubes were then loaded with the CDOM samples, and covered by thin PVC film (0.008 mm) to exclude dust and prevent contamination. The PVC film did not absorb light in UV and visible wavelengths.

For the natural solar radiation exposures, the filtered samples were placed in sealed quartz tubes (60 ml positioned horizontally), and submerged in a temperature-controlled water bath at 25°C. There were 50 samples: (triplicates ×8 times for the WDA and PDA CDOM, and 2 dark control samples for WDA CDOM). The two dark control samples (CK) of WDA CDOM were wrapped with several layers of aluminum foil, and placed in the exposure bath in an unshaded area. Every three sample was collected for analysis of the WDA and PDA CDOM in the evening. The dark control samples of river CDOM were collected at days 5 and 7. The water level was monitored during the incubation and analyzed concentrations were adjusted for evaporative losses.

### Ultraviolet (UV) radiation measurement

UV radiation (290–400 nm) was measured using a CUV3 radiometer (Kipp & Zonen Corporation, The Netherlands), located at the TLLER adjacent to the water bath used for the CDOM bleaching experiment; the CUV3 radiometer had a relative error <5%. The daily value of UV radiation was integrated from the minute values, and the cumulative UV radiation during the 9 days of the experiment was integrated from the day values.

### UV-Visible (UV-Vis) absorption measurements

Absorption spectra were obtained between 200 and 800 nm at 1 nm intervals, using a UV–Vis UV–2550PC spectrophotometer (Shimadzu) with matching 5 cm quartz cells. The slit width was 1 nm, and the wavelength scan rate was 210 nm min^-1^. Milli–Q water was used in the reference cell. Absorbance measurements at each wavelength (λ) were baseline corrected by subtracting the absorbance at 700 nm of the sample itself. Absorption coefficients were calculated by multiplying the corrected optical density by 2.303/*r*, where *r* is the cuvette path length in m. The detection limit of the optical density of the Shimadzu UV–2550PC spectrophotometer was 0.0001, thus the precision of the CDOM absorption coefficient was 0.005 m^-1^. In the present study, the concentration of CDOM is expressed using the absorption coefficient (m^-1^) at a wavelength of 254 m.

The spectral slope of the CDOM absorption curve (*S*) was calculated by non-linear regression over the 280–500 nm wavelength range according to the following equation [21]:
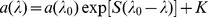
(1)where *a*(*λ*) is the absorption coefficient at wavelength *λ*, *a*(*λ*_0_) is the absorption coefficient at a reference wavelength *λ*_0_ of 440 nm, and *S* is the spectral slope (a measure of the decrease in absorption with increasing wavelength). *K* is a background parameter, which accounts for baseline shifts or attenuation due to factors other than CDOM. The spectral slope ratio (*S*_R_) was defined as the ratio of the spectral slopes of the shorter (275–295 nm) to the longer (350–400 nm) wavelength ranges [26].

The molecular size (*MS*; i.e., the mean molecular weight) of DOM was estimated from the ratio of the absorption coefficients at 250 and 365 nm (*a*(250)/*a*(365)), in which decreasing ratios indicate increasing molecular size [27]. The inverse relationship has been applied extensively to characterize the molecular size of dissolved organic matter in various environments [26], [28], [29], including humic like organic molecules extracted from atmospheric aerosol particles [30].

Photobleaching rates at 254 nm were quantified using the first-order kinetics equation with *a*(254) versus cumulative UV radiation (290–400 nm) measured.

### Fluorescence spectroscopy measurement

The fluorescence properties of CDOM were measured in cells with a 1-cm pathlength, using a Hitachi F-7000 fluorescence spectrometer (Hitachi High Technologies, Tokyo, Japan) with a 700-voltage xenon lamp at room temperature (20 ±2°C). The scanning ranges were 200–450 nm for excitation, and 250–600 nm for emission. Readings were collected in ratio mode (S/R) (the default mode of F-7000 fluorescence spectrometer) at 5 nm intervals for excitation and 1 nm interval for emission using a scanning speed of 2400 nm/min. There were negligible differences in the EEMs derived from the scanning speed of 2400 nm/min we used and EEMs derived from scanning speeds of 1200 nm/min used. The bandpass widths were 5 nm for both excitation and emission.

Water Raman scatter peaks were eliminated by subtracting a Milli-Q water blank from the EEMs. The spectra were corrected for instrumental response according to the procedure recommended by Hitachi (Hitachi F-7000 Instruction Manual). First, excitation was calibrated with Rhodamine B as standard (quantum counter), and using a single-side frosted red filter in excitation scan mode. Next the emission was calibrated with a diffuser in synchronous scan mode. The excitation and emission spectra obtained over the range 200–600 nm were applied internally by the instrument to correct the subsequent spectra.

The CDOM absorbance was used to correct the measured EEMs to eliminate the inner-filter effect, as detailed in our previous studies [8], [31]. Daily variations in fluorescence intensity were calibrated and normalized in quinine sulfate units (QSU), where 1 QSU is the maximum fluorescence intensity of 0.01 mg/L of quinine (qs) in 1N H_2_SO_4_ at the excitation wavelength (Ex; nm)/emission wavelength (Em; nm)  = 350/450 [32]. Rayleigh scatter effects were removed from the data set by excluding any emission measurements made at wavelengths ≤ excitation wavelength +20 nm, and at wavelengths ≥ excitation wavelength +280 nm. Zeros were added to the EEMs in the two triangle regions of missing data (emission wavelength ≤ excitation wavelength +20 nm, and ≥ excitation wavelength +280 nm). The contour figures of EEMs were drawn using Origin 8.5.

### PARAFAC modeling

The PARAFAC statistically decomposes the complex mixture of DOM fluorophores into individual components, without any assumptions about their spectral shape or number [33]. The combination of EEMs and PARAFAC has been widely applied to characterize DOM from aquatic environments [34]–[37], and in laboratory and mesocosm experiments [38]. The PARAFAC analysis in our study was performed in MATLAB using the DOMFluor toolbox for MATLAB, according to Stedmon and Bro [39]. We ultimately used excitation wavelengths from 220 to 450 nm, and emission wavelengths from 300 nm to 600 nm.

Two PARAFAC models were created, one for the WDA CDOM samples with 27 EEMs, and one for the PDA CDOM samples with 25 EEMs. An initial exploratory analysis was performed in which outliers were identified and removed from the dataset. Two WDA CDOM samples and three PDA CDOM samples were considered outliers and removed, either because they contained some instrument error or artifact or, more likely, because they were properly measured but were very different from the others (determined by examining the leverage using DOMfluor).

### Nutrient measurements

Total dissolved nitrogen (TDN) and total dissolved phosphorus (TDP) values were determined with the use of alkaline potassium persulfate digestion [40], followed by spectrophotometric analysis using the Shimadzu UV–2550PC spectrophotometer. Values for TDN and TDP were measured from day 0 to day 6 only.

### Statistical analyses

Statistical analyses including mean values and linear fitting were performed with Statistical Program for Social Sciences (SPSS) 17.0 software. Differences in parameters between the WDA and PDA CDOM were assessed with independent sample *t*-tests (*p*<0.05). Regression analyses were used to examine the relationships among different parameters. Significance levels are reported as not significant (*p*>0.05) or significant (*p*<0.05).

## Results

### TDN and TDP

**Figure 2:**
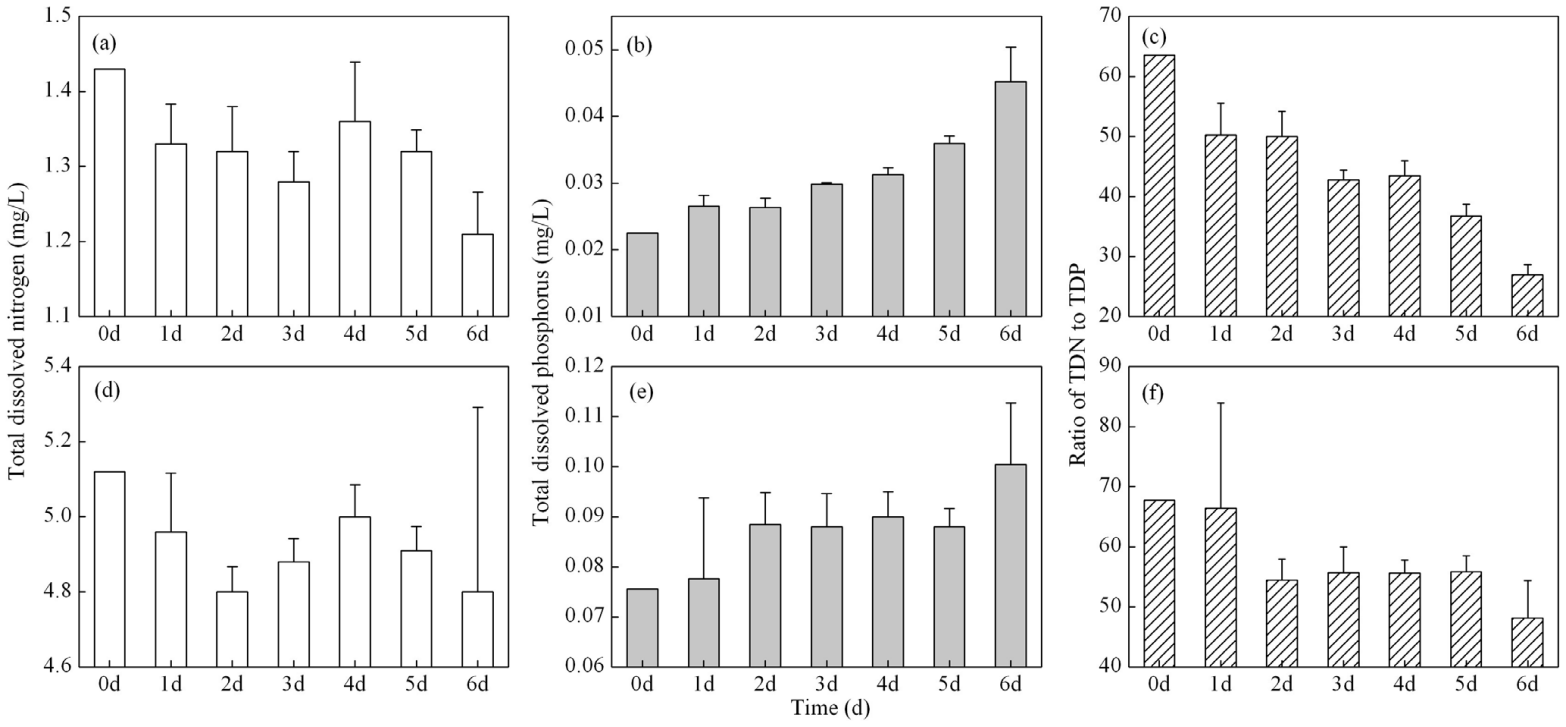
Temporal changes in total dissolved nitrogen concentration (TDN), total dissolved phosphorus concentration (TDP), and the ratio of TDN to TDP, for WDA (a–c) and PDA (d–f) CDOM samples exposed to natural solar radiation. Except for the initial value (0d), each measurement is an average of three samples (true replicates). Error bars indicate standard deviation.

The TDN concentrations decreased from 1.430 to 1.210±0.056 mg/L for WDA CDOM, and from 5.120 to 4.800±0.492 mg/L for PDA CDOM after 6 days exposure respectively (Fig. 2a, 2d), which were 84.6% and 93.7% of the initial values. The decrease was due to the release of ammonia into the atmosphere from TDN.

In contrast, during incubation exposure to natural solar radiation, TDP concentrations increased for both WDA and PDA CDOM (Fig. 2b, 2e). For WDA and PDA CDOM samples, TDP concentrations increased from 0.0225 to 0.0452±0.0052, and from 0.0756 to 0.1004±0.0123 mg/L after 6 days exposure, respectively, which were 200.9% and 132.8% of the initial values. The decrease in TDN and increase in TDP of the WDA CDOM sample were higher than those in the PDA CDOM sample.

The ratio of TDN to TDP for WDA CDOM was significantly lower than that for PDA CDOM (*p*<0.001, one-way ANOVA), showing that PDA CDOM sample had a higher percentage phosphorus concentration of CDOM than did the WDA CDOM sample. The ratio of TDN to TDP decreased with the exposure time for both WDA and PDA CDOM, due to the contrast variation trend. The ratio of TDN to TDP decreased from 63.56 to 26.91±1.76 for WDA CDOM, and from 67.73 to 48.13±6.25 for PDA CDOM after 6 days exposure, respectively, which were 42.34% and 71.07% of the initial values (Fig. 2c, 2f).

### CDOM photobleaching kinetics

**Figure 3:**
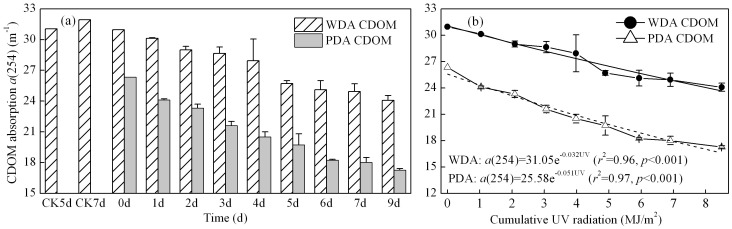
Temporal changes in CDOM absorption coefficients *a*(254) (a), and the first-order kinetics fitting of *a*(254) *vs* UV radiation cumulative value (b), of WDA and PDA CDOM samples. Error bars indicate standard deviations

Exposure of CDOM to natural solar radiation considerably reduced absorption for WDA and PDA CDOM (Fig. 3a). After 9 days exposure, CDOM absorption at 254 nm *a*(254) decreased to 24.08±0.47 and 17.27±0.14 m^-1^, with the loss of 6.88 and 9.07 m^-1^, for WDA and PDA CDOM respectively, which was only 78% and 66% of the initial values at day 0.

There was almost no difference in *a*(254) of the control samples at days 5 and 7 for WDA CDOM compared to the initial value (Fig. 3a), indicating no bacterial removal of CDOM. Although we did not set up a PDA control sample in the experiment, a supplementary experiment of PDA control sample did not show the marked variation in the CDOM absorption coefficient at days 2 and 5.

The WDA and PDA CDOM photobleaching experiments both produced *a*(254) statistically significantly fits for the first-order kinetics equation with time (not shown), and the UV radiation cumulative value (Fig. 3b). Due to the slight difference of natural solar ultraviolet irradiation from day 0 to day 9, cumulative UV radiation was used to fit *a*(254) decreases, and gives a reasonable and significant result (Fig. 3b). The photobleaching rate coefficient of WDA CDOM (0.032 m/MJ) was lower than that observed for the PDA CDOM (0.051 m/MJ).

### CDOM composition change

**Figure 4:**
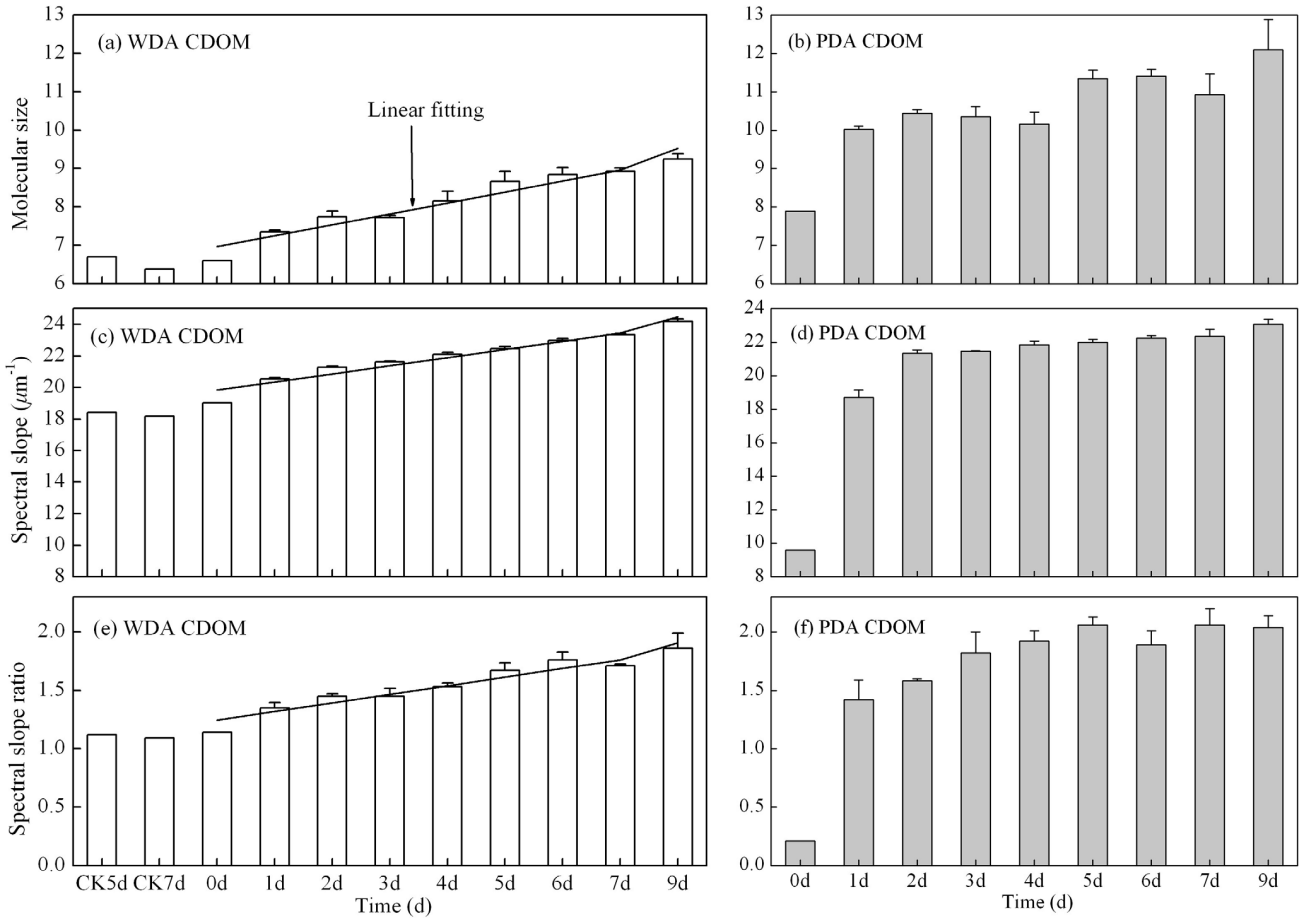
Temporal changes in three parameters of CDOM composition: molecular size (*MS*), spectral slope (*S*), and spectral slope ratio (*S*_R_) of WDA (a, c, e) and PDA (b, d, f) CDOM samples.

The three parameters of molecular size (*MS*), spectral slope (*S*) and spectral slope ratio (*S*_R_) have been widely used to examine changes in CDOM source, composition and degradation [26]. During our experiment, the values of each of these three parameters increased for both WDA and PDA CDOM (Fig. 4).

However, the nature of the increases differed according to the source of CDOM: in the WDA CDOM, *MS, S* and *S_R_* each increased gradually, whereas in the PDA CDOM increased initially very rapid. For the WDA CDOM, the gradual increases in *MS*, *S* and *S*_R_ with irradiation time from day 0 to day 9 were from 6.60 to 9.24±0.15, 19.04 to 24.18±0.18 µm^-1^, and 1.14 to 1.86±0.13 respectively (Fig. 4a, 4c, 4e). The significant linear increases in the slopes of *MS*, *S* and *S*_R_ were 0.284, 0.514 and 0.074. For PDA CDOM, at the start of the irradiation, the values of *MS*, *S* and *S*_R_ were 7.89, 9.6 µm^-1^ and 0.21, and these increased rapidly over the first day of the experiment to 10.02±0.09, 18.69±0.46 µm^-1^, and 1.42±0.17 respectively, and then gradually increased to 12.10±0.78, 23.07±0.29 µm^-1^ and 2.04±0.10 by day 9 (Fig. 4b, 4d, 4f).

The *MS* of WDA CDOM was significantly lower than that of the PDA CDOM (*p*<0.001, One-Way AVOVA); however, there were no significant differences in either *S* or *S*_R_ between the WDA and PDA CDOM. Thus, the *MS* was the better index by which to distinguish the WDA and PDA sources of CDOM.

**Figure 5:**
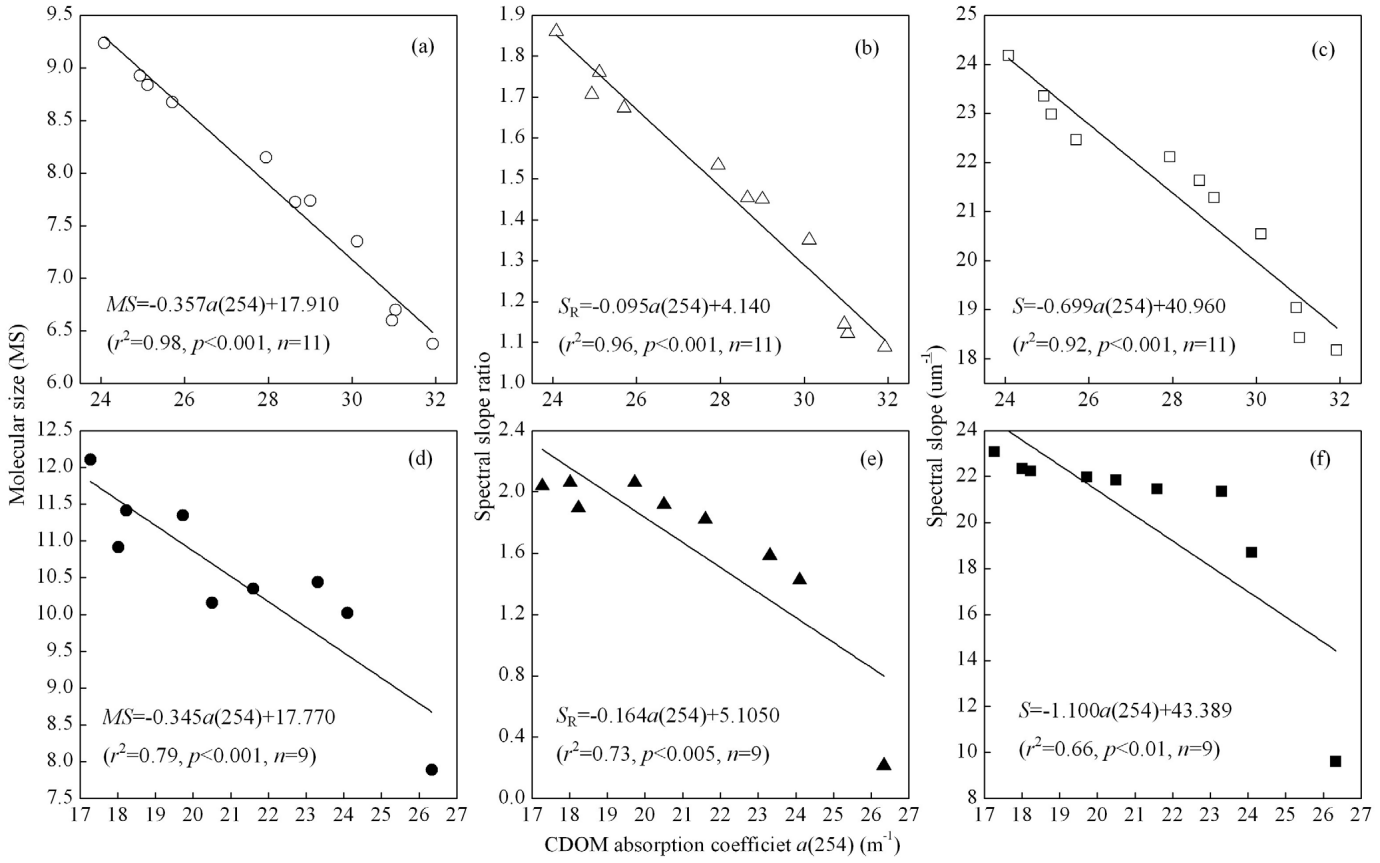
Correlations between CDOM absorption coefficient *a*(254) and molecular size *MS*, spectral slope ratio *S*_R,_ and spectral slope *S,* for WDA CDOM (a–c) and PDA CDOM (d–f).

There were significant negative correlations between *a*(254) and (i) *MS* (*r* ^2^ = 0.98, *p*<0.001 for WDA CDOM, and *r* ^2^ = 0.79, *p*<0.001 for PDA CDOM) (Fig. 5a, 5d), (ii) *S*_R_ (*r* ^2^ = 0.96, *p*<.001 for WDA CDOM and *r* ^2^ = 0.73, *p*<0.005 for PDA CDOM) (Fig. 5b, 5e), and (iii) *S* (*r* ^2^ = 0.92, *p*<0.001 for WDA CDOM and *r* ^2^ = 0.66, *p*<0.05 for PDA CDOM) (Fig. 5c, 5f). For PDA CDOM, the rapid variation of *MS*, *S*_R_ and *S* at day 1 of the experiment caused the scattering of the linear relationship. Thus, significant variations in CDOM composition and molecular size accompanied the decrease of CDOM absorption during WDA and PDA CDOM photobleaching.

### EEMs characteristics of CDOM

**Figure 6:**
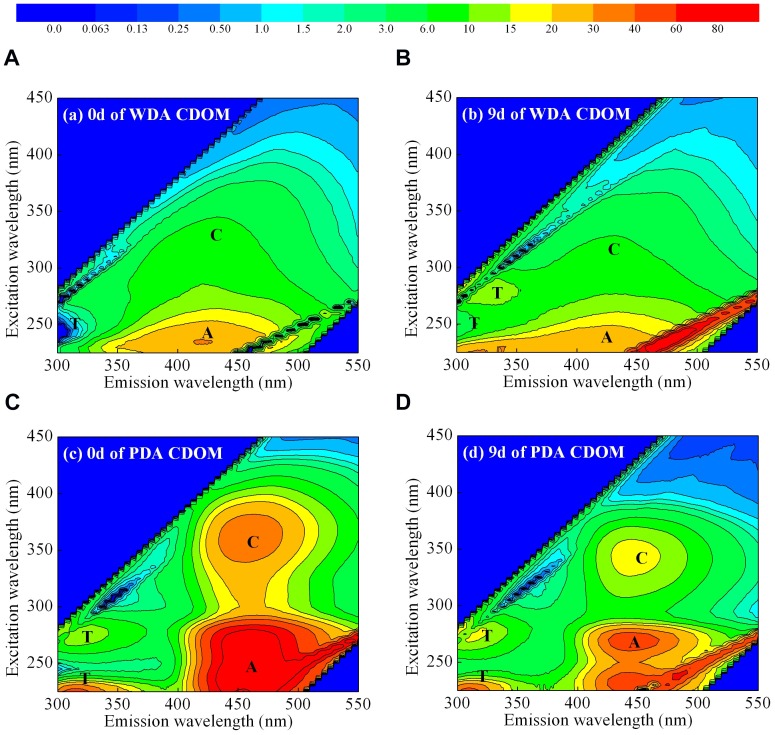
Examples of EEMs for days 0 and 9 for WDA CDOM (a, b), and for PDA CDOM (c, d), (b, d: data pooled for three parallel samples). Fluorescence is in QSU units. Peaks A and C represent terrestrial humic acids fluorescence, and peak T represents tyrosine fluorescence.

The four measured EEMs, at days 0 and 9 for both WDA and PDA CDOM (days with lowest and highest *a*(254) respectively, see Fig. 3a), are shown in Fig. 6. For WDA and PDA CDOM, both UV and visible humic-like fluorescence was prominent, as were peaks centered on (Ex/Em) 280/320 nm. The latter peaks are often attributed to “protein-like” fluorescence (for example of the aromatic amino acids tryptophan and tyrosine [41]), however a number of phenolic materials also fluoresce strongly in this region [42]. The common structure giving rise to the fluorescence in this region is the single aromatic ring [43]. Because no discrete chemical measurements of phenols or aromatic amino acids were made for this study, we refer to this region as “aromatic-like fluorescence”. For WDA CDOM, the first humic-like fluorescence peak was in the ultraviolet range (Ex_max_ = 225–250 nm, Em_max_  = 400–450 nm), and the second was in the visible range (Ex_max_  = 300–350 nm, Em_max_  = 400–450 nm); these peaks were originally designated as A and C, and exhibit fluorescence common to terrestrial humic substances [41]. For PDA CDOM, the similar humic-like fluorescence peaks were observed with the longer Ex_max_ (240–275 nm and 325–375 nm) and Em_max_ (425–475 nm).

Photobleaching decreased the humic-like fluorescence intensity but increased the aromatic-like fluorescence intensity and changed the positions of the peaks in both the WDA and PDA CDOM sources. The fluorescence intensity of the aromatic-like fluorescence peak (Ex_max_  = 225–240 nm, 260–280 nm and Em_max_  = 300–340 nm) (labeled T in Fig. 6) increased after 9 days exposure for both the WDA and PDA CDOM (Fig. 6). The fluorescence intensity of peak T (Ex_max_  =  225–240 nm and Em_max_  =  300–340 nm) increased 247.3% for the WDA CDOM. The Ex_max_ peaks of the visible humic-like fluorescence peak shifted toward shorter wavelengths for WDA and PDA CDOM during course of the degradation experiment, and this was more marked in the PDA CDOM than in the WDA CDOM (Fig. 6c–d).

### PARAFAC components

**Figure 7:**
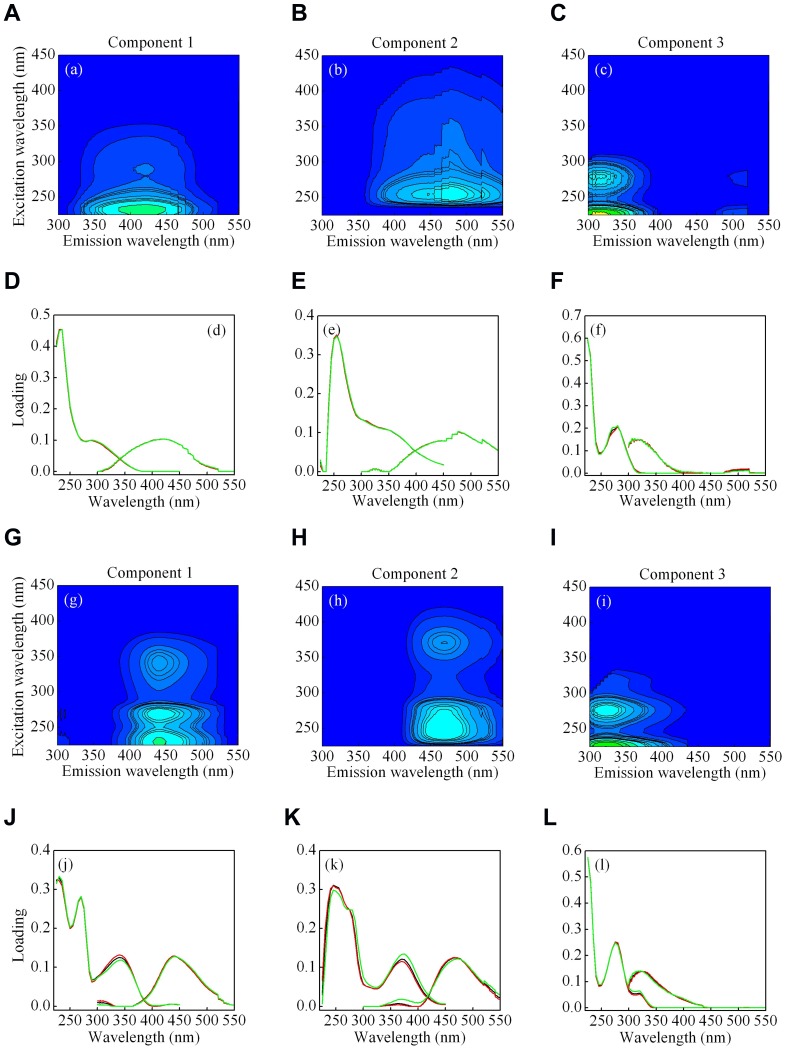
The PARAFAC model output showing fluorescence signatures of the three fluorescent components for the WDA (a–c) and PDA CDOM (g–i), and the contour plots presenting the spectral shapes of excitation and emission for the WDA (d–f) and PDA (j–l) CDOM. The line plots present split-half validation results; excitation (left) and emission (right) spectra were estimated from two independent halves of the dataset (red and green lines), and the complete dataset (black lines). A perfect validation is obtained if loadings from the two halves are identical.

Three fluorescent components were identified in PARAFAC fit for fluorescence properties in both WDA and PDA CDOM, based on the split-half validation procedure (Fig. 7). The largely overlapping excitation and emission loadings of the three components, modeled on each half of the dataset, and on the whole dataset, are shown in Fig. 7d–7f, 7j–7l. All three fluorescent components had single emission maxima, and single or multiple excitation maxima, denoting organic fluorophores, or groups of fluorophores exhibiting similar fluorescence. The excitation and emission characteristics of the CDOM fluorescent components we identified are given in Table 1, together with examples of matching components identified by other researchers who have modeled CDOM EEMs in aquatic environments using the PARAFAC model.

**Table 1:** Spectral characteristics of excitation and emission maxima of the three fluorescent components identified for the WDA and PDA CDOM by PARAFAC modeling, compared with those from previously identified sources.

Component No.	Ex_max_ (nm)	Em_max_ (nm)	Coble [41]; Coble et al. [44]	Other studies using PARAFAC	Description and probable source
C1-WDA	235 (290)	419	M peak: Ex_max_ = 290–310 and Em_max_ = 370–420	C3: Ex_max_ = 295 and Em_max_ = 398^1^C2: Ex_max_ = 315 and Em_max_ = 418^2^	Marine humic-like substances (biological degradation)
C1-PDA	230 (270, 340)	440			
C2-WDA	255	476	A peak: Ex_max_ = 230–260 and Em_max_ = 380–460C peak: Ex_max_ = 320–360 and Em_max_ = 420–480	C3: Ex_max_ = 270(360) and Em_max_ = 478^3^C4: Ex_max_ = 250(360) and Em_max_ = 440^4^C4: Ex_max_ = 255(370) and Em_max_ = 452^5^	Terrestrial humic-like substances or microbial degradation product of biological exudates; eutrophic surface waters
C2-PDA	245 (370)	470			
C3-WDA	≤225 (280)	311	B peak: Ex_max_ = 225–230(275) and Em_max_ = 305–310	C4: Ex_max_ = 275 and Em_max_ = 306(338)^1^C8: Ex_max_ = 275 and Em_max_ = 304^4^C1: Ex_max_ = 275 and Em_max_ <300^2^C7: Ex_max_ = 270 and Em_max_ = 299^6^	Aromatic-like fluorescence (tyrosine, phenol)
C3-PDA	≤225 (275)	323			

Secondary excitation band is given in brackets. Superscripts indicate the relevant reference citations: ^1^ [38]; ^2^ [35]; ^3^ [33]; ^4^ [45]; ^5^ [46]; ^6^ [36].

The three components we identified from the fluorescence spectra differed between the CDOM sources. For the WDA CDOM, the first two components (C1-WDA, C2-WDA) exhibited UV-like humic fluorescence similar to peak A (Table 1; Fig. 6). The third component (C3-WDA) exhibited aromatic-like fluorescence similar to peak T. This component was most like one of the components in the PDA CDOM (C3-PDA). The PDA CDOM PARAFAC model differed substantially in the first two components (C1-PDA, C2-PDA) from those of the WDA model (C1-WDA, C2-WDA). Each of the two PDA PARAFAC components exhibited strong dual excitation peaks, and intense red-shifted fluorescence peaks similar to the peak A and peak C regions.

For WDA CDOM, C1-WDA and C2-WDA exhibited excitation and emission maxima most similar to terrestrial sources [41], [44]. The peak fluorescence was blue-shifted to the UV, and had tailing emission into the red. By contrast, the distinctive dual peaks in the C1-PDA and C2-PDA exhibited fluorescence produced by microbes growing on amino sugars [47], phytoplankton productivity [48], and also were produced and altered by microbial reprocessing during a mesocosm experiment with CDOM produced by plankton [38]. Moreover, these components were very similar to components modeled for eutrophic lakes and estuaries [46], [49], [50].

For both WDA and PDA CDOM, component 3 (C3-WDA, C3-PDA) had excitation and emission characteristics similar to those of an autochthonous aromatic-like amino acid, similar to tyrosine, with excitation maxima at ≤225 nm (280 nm) and emission maximum at 311 nm [36], [37], [44]. This component likely represented autochthonous CDOM. Component 3 of PDA CDOM had similar excitation maxima at ≤225 nm (280 nm) to that of WDA CDOM, but a longer emission maximum at 323 nm.

**Figure 8:**
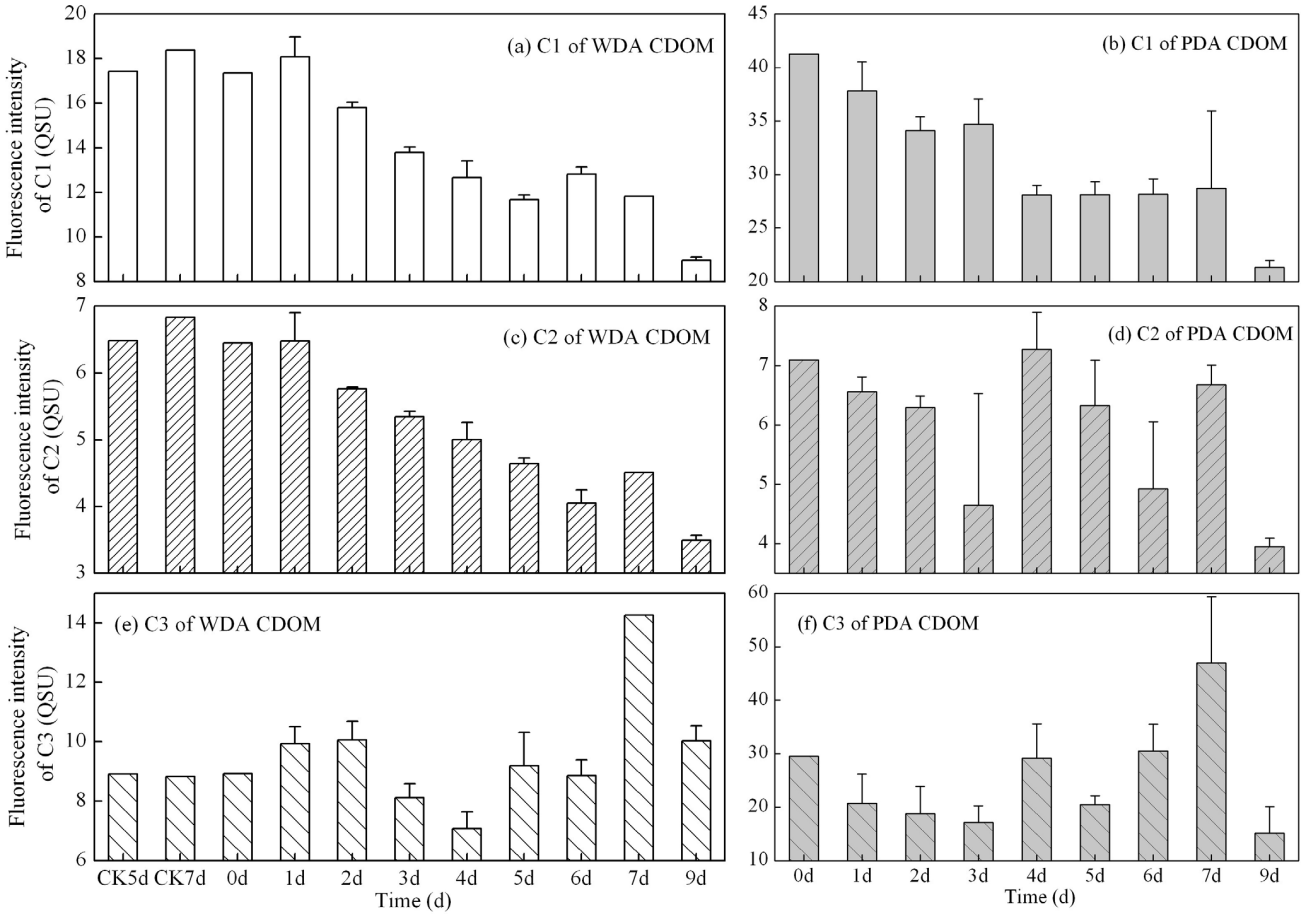
Temporal variations in intensity of CDOM fluorescence components for WDA (a–c) and PDA (d–f) CDOM samples. For comparison, the control samples (CK5d, CK7d) after 5d and 7d for the WDA experiment are shown. Error bars indicate standard deviation.

Temporal variations in the mean fluorescence intensity of the three components derived by the PARAFAC model during the degradation experiment are shown in Figure 8. At the beginning (0 d) of the photobleaching experiment, the fluorescence intensity of components 1 and 2 was the highest. For the control WDA CDOM samples, there was almost no difference in the fluorescence intensity at days 5 and 7 compared to the initial value for all three components (Fig. 8a, 8c, 8e), indicating no bacterial removal of CDOM. For WDA CDOM, the photobleaching exposure to natural solar radiation resulted in components 1 and 2 gradually decreasing to values significantly lower than the initial values (*t*-test, *p*<.001); after 9 days exposure, the fluorescence intensity of components 1 and 2 was only 51.56% and 54.12% of their initial values at day 0, respectively. For PDA CDOM, the photobleaching exposure to natural solar radiation resulted in component 1 gradually decreasing to a value significantly lower than the initial value (*t*-test, *p*<0.001). In contrast, there was no consistent decreasing trend for component 2 of PDA CDOM. Rather, this component oscillated in intensity, first decreasing, then increasing, and finally decreasing, during the course of the exposure experiment (Fig. 8d).

The fluorescence intensity of component 1 in both types of CDOM decreased more rapidly than did CDOM absorption *a*(254), indicating that fluorescent dissolved organic matter (FDOM) was more easily degraded by solar radiation than non-FDOM. The fluorescence intensity of component 3 increased after 9 days exposure for WDA CDOM (Fig. 6b, Fig. 8e). However, the C3 intensity oscillated during the exposure, initially decreasing during the first 3–4 days, then increasing markedly after 7 days, then finally decreasing again after 9 days (Fig. 8e, 8f). This behavior was similar to component 2 in the PDA CDOM (Fig. 8d).

## Discussion

### Photobleaching mechanism of generating nutrients and its implications

Contrasting with the abundant information on photobleaching caused CDOM absorption decrease and dissolved organic carbon loss [13], [19], [51], [52], relatively little is known about the photoinduced transformations of nitrogen and phosphorus in natural waters [3], [20]. The limited information on the photoinduced transformations of nitrogen and phosphorus indicates that these transformations are complex, as evident by the contradictory results. While some studies have observed net ammonium photoproduction in natural waters to supports hetero- and autotrophic production [3], [15], [20], there are many reports that show either no photoproduction or a photoinduced loss of ammonium [53], [54].

Our results showed a marked decrease in TDN concentration under natural sunlight exposure for both WDA and PDA CDOM, indicating photoproduction of ammonium and the release of ammonia into the atmosphere from TDN. Previous similar studies have all showed an increase in ammonium concentration accompanying the decrease of TDN due to photoammonification [3], [55]. The proposed mechanism of TDN decrease and ammonium photoproduction was that the photoinduced breakdown of CDOM forms ammonium ion and release ammonia into the atmosphere. This behavior has been observed in humic waters [56]. In addition, the changes in the TDN concentrations, and the ratio of ammonium ion to ammonia may also be related to the pH changes in the experiment. The low pH may result in the reduction of ammonia, increasing the formation of ammonium ions in water. Since we did not measure pH, we cannot assess the effect of pH on the ratio of ammonium ion to ammonia during the photodegradation process. The exact reason for the increase of TDP in both WDA and PDA CDOM samples in the exposed experiment was unknown, due to the lack of additional measurements. A possible explanation is that the photodegradation of humic substances caused the partial release of TDP (likely as dissolved organic P) via the disruption of iron-P-humic complexes [57]. The effect appears not to be widespread across lake types [58], thought phosphate photoproduction appears to be more frequently observed in humic-rich lakes than in other lake types [59]. Our results extend the photoproduction of P to autochthonous sources of CDOM, because our PDA source also produced appreciable amounts of TDP. Thus photobleaching has an important effect on phosphorus biogeochemistry in lakes.

Due to the converse dynamics of TDN and TDP during the photoinduced transformation process, TDN∶TDP ratio decreased significantly both for WDA and PDA CDOM, which partially explained the seasonal dynamics of the TDN∶TDP ratio in Lake Taihu. During winter and spring, TDN∶TDP ratios ranged from 98 to 124∶1 in Meiliang Bay, but in summer, this ratio dropped below 28∶1. The ratios in the central lake varied between 92∶1 and 158∶1 during winter and spring, and then declined in late summer to early fall below 36∶1 [60]. In addition to the phosphorus internal recycle driven by microbial and physical-chemical condition (pH), higher nitrogen loss via denitrification in summer, photoinduced transformation of CDOM was also the poential drive factor of the nitrogen to phosphorus ratio. The photoinduced transformation of CDOM is the largest during summer when solar irradiance is the highest, thus causing the summer decrease of TDN∶TDP ratio in the natural lake water. The decrease of TDN∶TDP ratio in summer, close to the Redfield ratio (7∶1), is favorable to phytoplankton growth and supports the magnitude and duration of algal blooms in Lake Taihu.

**Figure 9:**
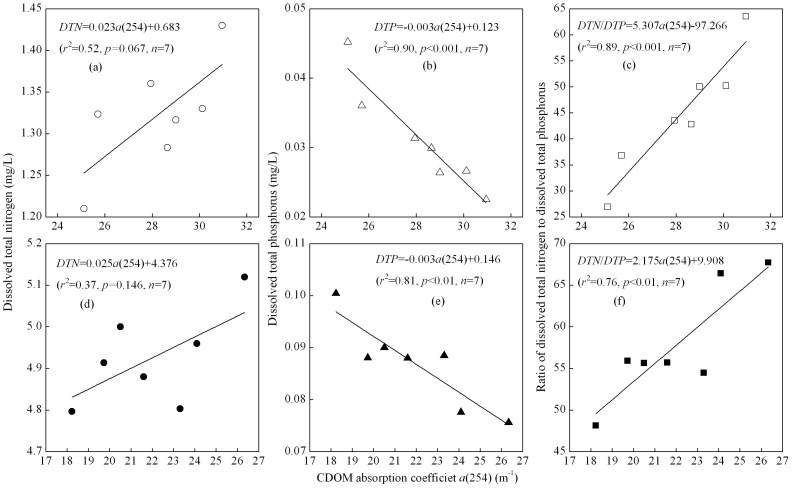
Linear relationships between CDOM absorption coefficient *a*(254) and total dissolved nitrogen concentration (TDN), total dissolved phosphorus concentration (TNP), and the TDN∶TDP ratio, for WDA (a–c) and PDA (d–f) CDOM.

There was a significant positive linear relationship between *a*(254) and TDN∶TNP ratio, and a significant negative linear relationship between *a*(254) and TDP concentration (Fig. 9). There was a positive (but not statistically significant) linear relationship between *a*(254) and TDN concentration (Fig. 9). These relationships indicated a coupling process between CDOM absorption and nutrient concentrations, further confirmed that the remineralization of CDOM by photobleaching played an important role in nitrogen and phosphorus cycling and phytoplankton growth in the lake.

### CDOM photobleaching and composition change

Numerous compounds with diverse chemical structures contribute to CDOM, and it is likely that these compounds would vary in their susceptibilities to photodegradation. A steep *S* and large *MS* values indicated low molecular weight material or decreasing aromaticity, and a shallow *S* and small *MS* values indicated CDOM with a high molecular weight and high aromatic content [26], [27], [51]. The increase of *S* and *MS* with irradiation time for both WDA and PDA CDOM suggested a decrease of molecular weight, confirming that photodegradation could degrade CDOM molecules and lead to a decrease in molecular weight, which has been observed in other similar studies [28]. In addition, Helms et al. [26] also showed that *S*_R_ for high molecular weight fractions of DOM were generally lower than those for the corresponding low molecular weight fractions. Further, Helms et al. [26] found that *S*_R_ generally increased upon irradiation, indicating the decrease of molecular weight due to photodegradation. Our results that *S*_R_ increased with irradiation time therefore suggests that molecular weight decreased for both PDA and WDA CDOM. For PDA CDOM, a sharp increase of *MS*, *S* and *S*_R_ after 1 day indicated a significant change of CDOM composition and molecular weight. The PDA CDOM included a very labile portion, which was easily photobleached by natural solar radiation.

### Characteristics of fluorescence components

An important outcome of our study was that allochthonous and autochthonous sources of CDOM to Lake Taihu were optically different. Application of PARAFAC to the CDOM from our photobleaching experiment revealed that three components modelled aquatic CDOM, namely two humic-like components and an aromatic-like component for the WDA. Previous studies reported the presence of 3 – 13 components in DOM from diverse aquatic environments [8], [34]–[36], [46]. The wide variation in the number of components calculated by PARAFAC models for diverse aquatic environments likely reflects the mixing and spectral alteration of a few primary fluorescent components.

The spectral characteristics of the three components identified by modeling each DOM source (WDA vs PDA) were similar to those of CDOM previously reported in other aquatic environments using the PARAFAC model [7], [8], [31], [33], [34], [36], [38], [45]. For both allochthonous and autochthonous CDOM sources (WDA and PDA, respectively), PARAFAC modeled CDOM fluorescence components which contained humic-like signatures, having red-shifted emission, and indicating highly conjugated or condensed structures [61]. Further, both CDOM sources contained a blue-shifted component 3 (though with different Ex/Em features, Table 1), exhibiting fluorescence similar to phenols or to tryptophan or tyrosine residues [43].

Despite these similarities, there was a clear distinction between the two sources. Components 1 and 2 in the WDA model exhibited features very similar to humic substances exported from soils [62], which was expected. However, we also found similar, yet distinct, red-shifted emission released during senescence and microbial degradation of phytoplankton. Component 1 from the PDA model had Ex/Em maxima which were very similar to a photolabile CDOM component modeled with PARAFAC on a mixotrophic lake in the US Great Plains that receives substantial nutrient input [49]. Further, component 2 from the PDA model had Ex/Em maxima which closely resembled components modeled by PARAFAC for nutrient-sensitive waters [46], [49], [50]) and which also resembles DOM fluorescence produced from algae cultures [48]. To our knowledge our present study is the first to show a side by side comparison of CDOM from nominally soil-derived and phytoplankton-derived sources, with clear implications for the underlying DOM biogeochemistry in eutrophic aquatic ecosystems.

**Figure 10:**
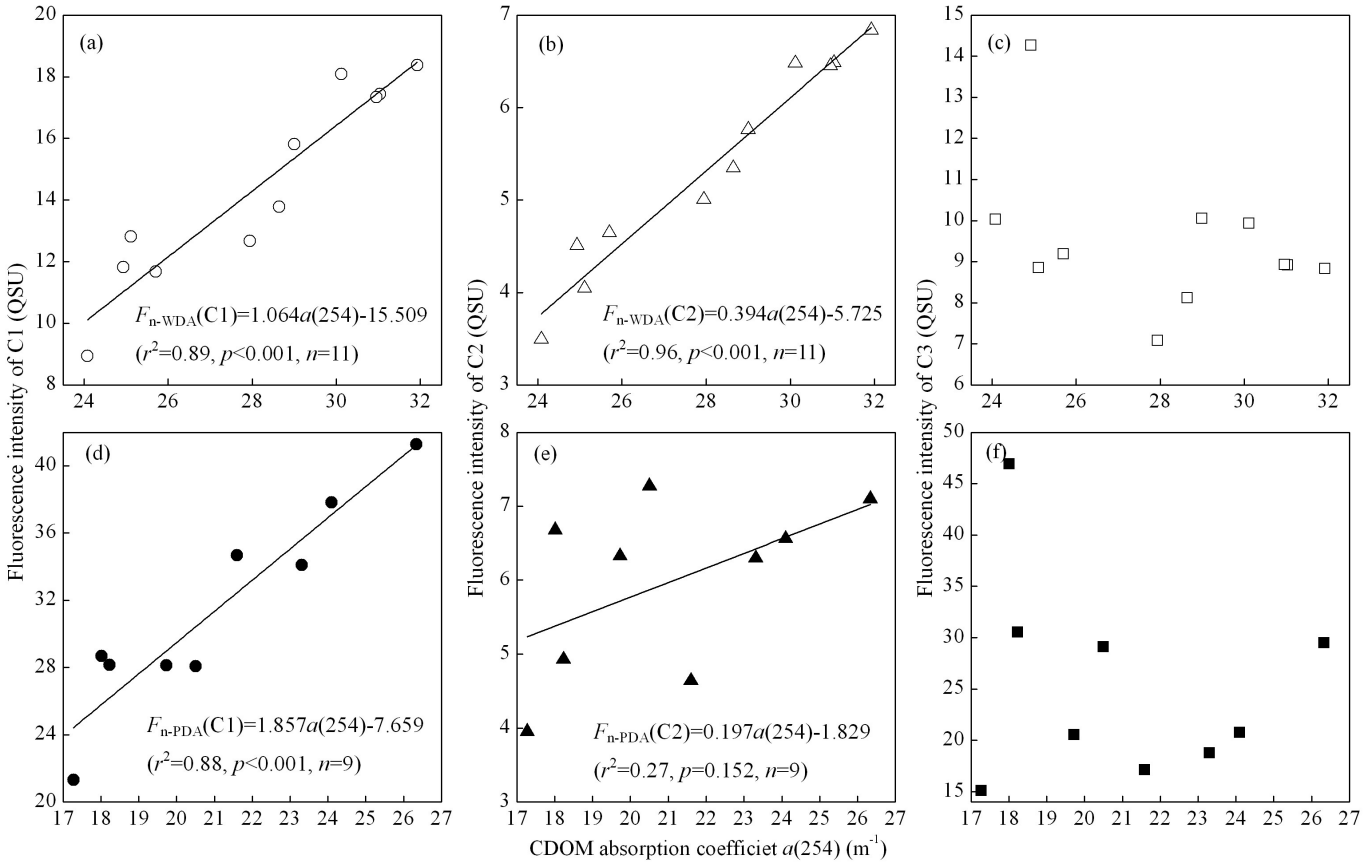
Linear relationships between the CDOM absorption coefficient *a*(254) and the fluorescence intensity for three fluorescence components for WDA (a–c) and PDA (d–f) CDOM.

One implication is the humic-like fluorescence materials, regardless of source, could be rapidly and easily photobleached. For WDA and PDA CDOM, there were positive relationships between *a*(254) and the fluorescence intensity of components 1 and 2 (Fig. 10a, 10b, 10d).

In contrast, there was no positive relationship between *a*(254) and the fluorescence intensity of component 3 for either WDA or PDA CDOM (Fig. 10c, 10f), indicating that the aromatic-like fluorescence, very likely attributable to residues of tryptophan and/or tyrosine, could not be photobleached or only slightly photobleached. Some studies have shown that the protein-like fluorescence materials (peak T) were not photobleached rapidly. For example, Stedmon et al.[3] reported that fluorophore 5 (corresponding to proteinaceous T peak) photobleached less than did fluorophores related to humics in Baltic Sea samples. Similarly, photobleaching of the T peak was also less intense than photobleaching of humic fluorophores in *Juncus* leachate [63]. In addition, Berto et al. [64] reported that irradiation would caused a significant increase of tyrosine absorption, which supported the protein-like fluorescence peaks strength increase in Fig. 6, indicating the transformation of the humic-like fluorescence substance to the protein-like fluorescence substance. Of course, we also note that some studies reported that the proteinaceous T peak was readily photodegraded on exposure to solar radiation [19], [47]. The photobleaching response difference of the aromatic protein-like fluorescence possibly was due to the structural arrangement by which tryptophan or tyrosine was bound to larger proteins [43].

### Implication of CDOM photobleaching in lake waters

Many studies of the decomposition of CDOM have been conducted under natural or simulated solar radiation without microbes, to determine the rates and extent of photodegradation. These studies have been particularly useful for understanding the mechanisms of photoinduced processes to CDOM decomposition.

Terrestrially-derived DOM produced during the decomposition of vascular plant tissue is generally considered to be more chemically complex than is the CDOM produced by phytoplankton, marsh and microbial activity within the water column [17], [51]. Consequently, CDOM derived from terrestrial sources is often composed of myriad aromatic functionalities which strongly absorb UV, and are susceptible to photoinduced destruction [65]. Thus, we expected to find higher photobleaching rate coefficients for samples collected in the river since they were most likely to contain a higher proportion of terrestrially derived CDOM. However, our result shows that PDA CDOM has a higher photobleaching rate coefficient (0.051 m/MJ of PDA CDOM *vs* 0.032 m/MJ WDA CDOM). A possible explanation for the lower photobleaching rate coefficient of WDA CDOM than PDA CDOM is that the surface WDA CDOM pool had already been exposed to an extended period of summer time solar radiation prior to our sampling in early August, reducing the photoreactivity of the CDOM pool across our study region. A similar result had been observed in northern Gulf of Mexico shelf waters [66]. Another possibility is that phytoplankton leaches highly photolabile CDOM, which enriched some substances with fluorescence properties similar to C2 for the PDA CDOM. These microbial/algal origin autochthonous fulvic acids rich in fluorophores were rapidly decomposed by natural sunlight [67]. It had recently been found that algal-derived DOM is a more efficient photoinduced substrate than is allochthonous DOM [13], [68]. However, at present such substances are unknown, which would be the focus for future research challenges.

The results from our photobleaching experiments could have significant implications for productive lakes. We now estimate the relative contribution of the CDOM photobleaching by sunlight to the total CDOM in Lake Taihu, to elucidate the importance of photobleaching in CDOM biogeochemistry. The monthly mean daily UV radiation cumulative value in summer (June-August) in Lake Taihu was 0.784 MJ/m^2^ based on UV radiation measurement in TLLER. Thus, the contribution of CDOM photobleaching *a*(254) was 0.77–1.00 m^-1^ based on the *a*(254) photobleaching rate of 0.032 m/MJ for WDA CDOM, and 0.051 m/MJ for PDA CDOM in our photobleaching experiment, accounting for 3.9%–5.1% of the monthly mean value of 19.54 m^-1^ the lake [21]. The result showed that 3.9%–5.1% CDOM at the water surface was photobleached and mineralized every day in summer in Lake Taihu.

In our study, bacteria were removed in order to elucidate the effect of photobleaching on CDOM mineralization. In the actual natural conditions of the lake waters, the sunlight helps to break down many complex polymeric structures of CDOM [49], [51], and make them more labile for microbial uptake. Microbial decomposition is ubiquitous and may remineralize CDOM to release nutrients especially nitrogen to stimulate higher trophic levels and autotrophic plankton [15]. In Lake Taihu, due to the frequent algal bloom, the inorganic nutrients which phytoplankton could absorb and utilize were often incorporated into the algal biomass during the algal bloom period [60], [69]. Therefore, the degradation and mineralization of PDA CDOM resulting from photobleaching and microbial decomposition to produce inorganic nutrients, provided a new source of nutrients which could play a significant role in helping sustain blooms during the summer when availability of nitrogen was a key growth-limiting factor for phytoplankton growth [60]. However, the extent to which photobleacing provides nutrients, relative to other sources (riverine inputs and internal release) remains unknown, and needs further study. More systematic control experiments to assess the effects of the production inorganic nutrients from photobleaching and microbial decomposition on phytoplankton growth would be performed in future studies. In addition, we need to add other sources CDOM to compare and assess the photoinduced degradation dynamics of CDOM in the further study, considering that only two representative sources of CDOM and summer river WDA were included in the present study.
